# Primary Immune Deficiency: Patients’ Preferences for Replacement Immunoglobulin Therapy

**DOI:** 10.3389/fimmu.2022.827305

**Published:** 2022-02-04

**Authors:** Juan Marcos Gonzalez, Mark Ballow, Angelyn Fairchild, Michael Chris Runken

**Affiliations:** ^1^ Department of Population Health Sciences, Duke University, Durham, NC, United States; ^2^ Division of Allergy & Immunology, Department of Pediatrics, Morsani College of Medicine, University of South Florida, Tampa, FL, United States; ^3^ Johns Hopkins All Children’s Hospital, Saint Petersburg, FL, United States; ^4^ Kenan-Flagler Business School, University of North Carolina at Chapel Hill, Chapel Hill, NC, United States; ^5^ Scientific & Medical Affairs, Global Health Economics and Outcomes Research, Grifols SSNA, Durham, NC, United States

**Keywords:** primary immune deficiency disorders, immunoglobulin replacement therapy, IVIg, SCIG, patient preferences, discrete-choice experiment

## Abstract

**Purpose:**

Immunoglobulin (Ig) replacement therapy is an important life-saving treatment modality for patients with primary antibody immune deficiency disorders (PAD). IVIG and SCIg are suitable alternatives to treat patients with PAD but vary in key ways. Existing evidence on patient preferences for Ig treatments given the complexities associated with IVIG and SCIg treatment is limited and fails to account for variations in preferences across patients. For this reason, we sought to evaluate PAD patient preferences for features of IVIG and SCIg across different patient characteristics.

**Materials and Methods:**

119 PAD patients completed a discrete-choice experiment (DCE) survey. The DCE asked respondents to make choices between carefully constructed treatment alternatives described in terms of generic treatment features. Choices from the DCE were analyzed to determine the relative influence of attribute changes on treatment preferences. We used subgroup analysis to evaluate systematic variations in preferences by patients’ age, gender, time since diagnosis, and treatment experience.

**Results:**

Patients were primarily concerned about the duration of treatment side effects, but preferences were heterogeneous. This was particularly true around administration features. Time since diagnosis was associated with an increase in patients’ concerns with the number of needles required per infusion. Also, patients appear to prefer the kind of therapy they are currently using which could be the result of properly aligned patient preferences or evidence of patient adaptive behavior.

**Conclusions:**

Heterogeneity in preferences for Ig replacement treatments suggests that a formal shared decision making process could have an important role in improving patient care.

## Introduction

Immunoglobulin (Ig) replacement therapy is an important life-saving treatment modality for patients with primary antibody immune deficiency disorders (PAD), especially those with antibody deficiency that account for approximately 50% of all types of primary immune deficiency disorders. The goal of treatment is to provide a broad spectrum of antibodies to prevent infections, inflammatory injury to vital organs like the lung, and chronic long-term complications.

Intramuscular gammaglobulin was first used in the early 1950s as replacement therapy until intravenous immunoglobulin (IVIG) was approved in 1981. This was a notable advancement since IVIG could essentially normalize the serum levels of IgG, and more productively protect patients from infection and even chronic lung disease. Clinical immunologists in Sweden took a different approach administering IVIG by the subcutaneous route. Gardulf et al. (1) and Ochs et al. (2) showed that the subcutaneous route for Ig replacement therapy, e.g. SCIg was safe, well tolerated, and effective in achieving adequate serum IgG levels. In a multicenter study of 165 patients with hypogammaglobulinemia receiving subcutaneous infusions (27,030 at home) a significant reduction in adverse systemic reactions was observed compared with intramuscular or intravenous administration. Although serious systemic reactions did not occur with SCIg, local tissue reactions did occur including swelling, soreness, redness, induration, itching, and bruising, but these were not serious and usually resolved with 48-72 hours. Thus, SCIg is a suitable alternative to IVIG and may present certain opportunities for optimizing at-home care for patients with PAD (3).

The SCIg products are 10%, 16.5% or 20% formulations; the 10% are products similar in composition to the IV product. Depending on the product, SCIg can be given biweekly, weekly or even more frequently as a subcutaneous push. The number of infusion sites varies from a single site to four sites depending on the product formulation (10% vs 20%), dosages, body weight of the patient and frequency (4).

A number of surveys have been published examining patients’ health-related quality of life (HRQoL) (5) and treatment satisfaction with IVIG and SCIg replacement therapy in PAD patients. Several studies have shown enhancements in HRQoL with various treatment options, but it has been acknowledged that there is also “substantial treatment burden” and the burden can vary between the IV and SC routes, and site of care (5).

Multiple reports have shown that most patients choose home-based Ig replacement therapy and switch from receiving IVIG in a hospital to IVIG administered by a travel nurse, or SCIg self-administered in a home based setting (6–8). However, some evidence suggests that patients’ perspectives could change with specific treatment experiences as Routes et al., (2016) found that about 88% of patients switched to IV administration at the hospital after 12 months of treatment (9).

Environmental and personal factors also can play a role in patients’ preferences for PAD treatments. During the COVID-19 pandemic, some patients with PAD experienced high levels of anxiety and poor HRQoL when receiving hospital-based infusions. Others feared supply shortages while being treated at home (10, 11). The patient’s job or lifestyle requirements also can affect their preferences, particularly if the patient must travel frequently (5). All of this highlights the importance of patients’ perspectives in the selection of treatment options.

Particularly because IVIG and SCig are largely equivalent in terms of efficacy, the appropriateness of these options for a specific patient may be a matter of preference, or the relative importance of the features of each administration option. Recent studies have formally elicited stated preferences for treatments given the tradeoffs associated with IVIG and SCIg. This research typically differs from HRQoL evaluation tools in that it decomposes the relative importance of treatment factors to understand which aspects matter most to patients. Among PAD patients, this evidence has been rather limited (12, 13). Mohamed et al. (12), reported on patient and parent preferences for Ig replacement therapy attributes. Both parents and patients found that Ig administration in the home was preferable, with monthly frequency of the treatment using fewer needle sticks. A shorter duration of the treatment was also desirable. This work, however, did not assess the relationship between individual patient characteristics and treatment preferences.

While the available evidence on patient preferences suggests that at-home self-administration is generally preferred by patients, this perspective on treatment type is likely not universal. To date, little to no attention has been given to explaining what factors may be associated with different perspectives on treatments. Understanding the association between patient characteristics and treatment preferences can help patients and clinicians evaluate treatment options in a more efficient and meaningful way (14). Furthermore, understanding variations in patient preferences could help reduce treatment burden among patients who are not currently matched with their own preferred alternative.

This study evaluates stated preferences for attributes of IV and SC routes of administration of Ig replacement therapy for PAD patients with differing personal characteristics. We look to collect evidence on the association of patient characteristics with route of treatment preferences. Specifically, to evaluate whether the patients’ age, years since diagnosis, gender and treatment experience made a difference in route of treatment choices.

## Materials and Methods

Adult patients with primary immune antibody deficiency who were members of the Immune Deficiency Foundation (IDF) or the Kantar Health Panel in the United States were invited to complete an online survey with a discrete-choice experiment (DCE). All respondents were required to have self-reported physician-diagnosis of PAD and to be able to provide consent.

The DCE was conducted following good-practice guidance (15). A DCE is a survey method that asks respondents to make choices between carefully constructed treatment alternatives where every treatment is described in terms of generic features called attributes. In our case, these attributes included route of administration, number of needle sticks required for administration, treatment frequency, administration times, and side effects duration. Treatment choices differ from each other based on experimentally-controlled variations in their performance under each attribute (attribute levels).

To define the study attributes, we conducted a 90-minute focus group with a convenience sample of six adult patients with PAD in the Atlanta metropolitan area. From the focus group, we collected feedback on the aspects of treatments for PAD that patients most liked and disliked. We also collected information on patients’ unmet needs, and treatment switching behavior and adherence. A comprehensive list of treatment-related aspects associated with the discussions during the focus group was defined based on participants’ feedback. At the end of the focus group, participants completed an attribute-prioritization exercise using Case-1 Best-Worst Scaling (16) to determine the treatment attributes that would be included in the DCE. The resulting attributes and attribute levels are summarized in **Table 1**.

**Table 1:** Attributes and attribute levels.

Attribute	Attribute Level
**How the treatment is administered**	Infusion under skin at home (no nurse)
	Infusion under skin at home (with nurse)
	Infusion under the skin at clinic (with nurse)
	Infusion into vein at home (with nurse)
	Infusion into vein at clinic (with nurse)
**How many needle sticks**	1 needle
	2 needles
	4 needles
**How often you take the treatment**	Once a month
	Twice a month
	4 times a month
**Administration time**	1 hour
	3 hours
	6 hours
**Time with headache and drowsiness**	None
	2 hours
	10 hours
	24 hours

**Figure 1:**
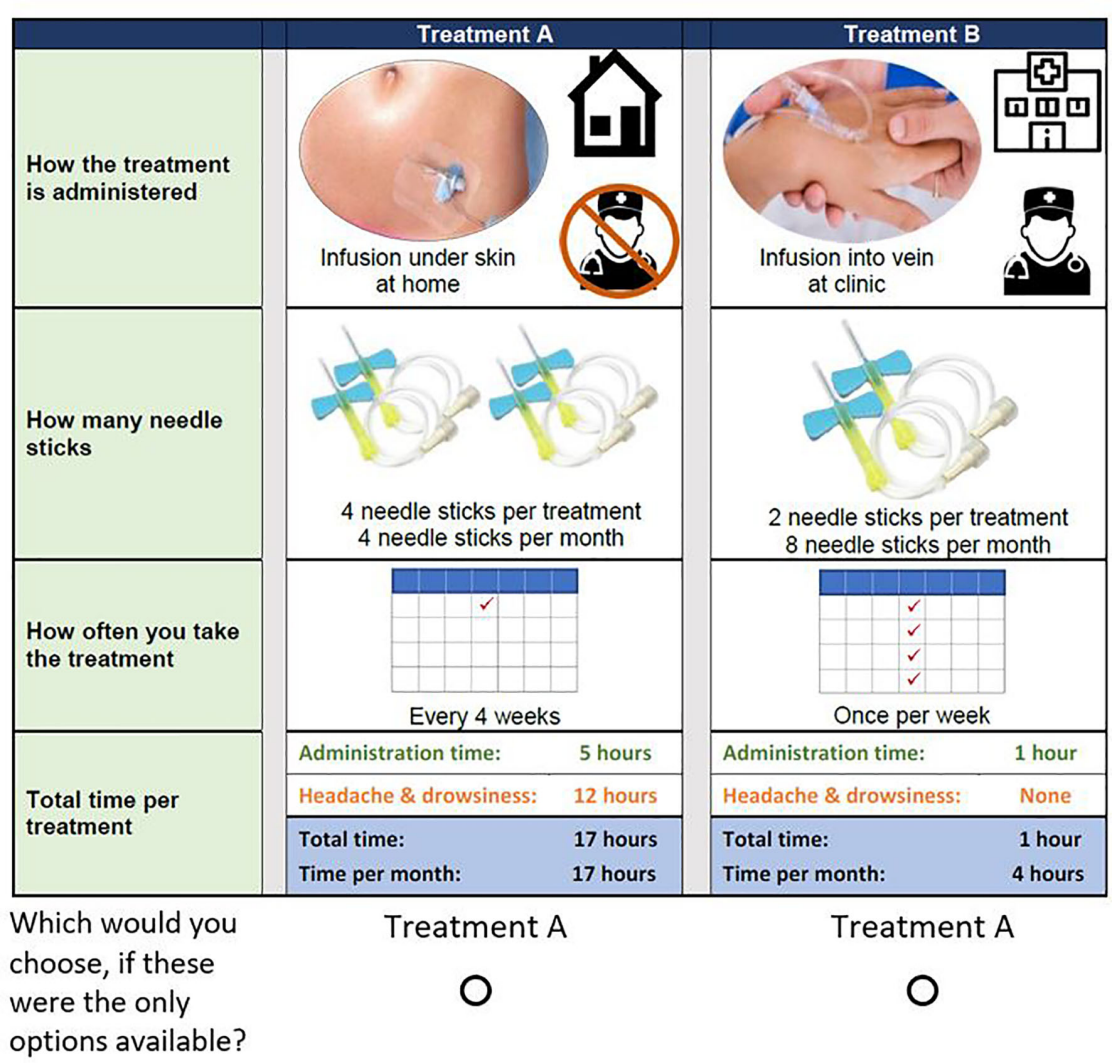
Example DCE choice question. Example question answered by study participants. Respondents were asked to answer 14 of these questions, each with different combinations of levels for each treatment attribute.

Based on the attributes and attribute levels selected, we developed a survey instrument with the input of preference researchers and clinical experts. The survey was pretested with a convenience sample of 5 adult patients with PAD, and 5 general-population respondents. Each individual interview was one-hour long and asked respondents to complete an online version of the survey instrument. During the pretest interviews, participants were asked to follow a think-aloud protocol. Respondents were asked to read the survey instrument out loud and were encouraged to articulate their thoughts related to survey information materials and questions. **Figure 1** presents the final choice question included in the survey.

The implementation of the DCE required the development of an experimental design with known statistical properties to populate the alternatives in the choice questions. We followed good-practice guidance on the development of the experimental design (17). Details on the experimental design can be found in **Appendix A**.

### Analysis

We first evaluated the validity of the DCE data based on commonly followed data quality checks, including response nonvariation in preferences (straight lining), attribute dominance, and attribute-comprehension questions (18). Additional information on these quality checks can be found in **Appendix A**. Respondents who were considered to be nonattentive based on these quality checks were excluded from the study sample. All mechanisms to address any observed quality issues were outlined prior to analysis.

After defining the final study sample, we followed good-practice guidance on the use of logit-based analysis to link patient responses to the tradeoffs required between the alternatives in the choice questions (19). Results from logit-based models produce preference weights in the form of log-odds (20). These weights reflect the average change in preferences for treatments with specific changes in attribute levels, all else equal. Additional details on the analysis of the preference data and the evaluation of variation in preferences can be found in **Appendix A**.

## Results

A total of 119 patients with PAD completed the survey instrument: 94 from the IDF and 25 from the Kantar Health Panel. **Table 2** presents the responses to the demographic and disease-experience questions included in the online survey instrument. The age distribution for respondents had a median of 51 years (range 18-77) Also, our sample was primarily female (87.4%). This is consistent with previous studies looking at quality of life outcomes among patients with PAD (8).

**Table 2:** Demographic characteristics and respondent disease experience.

	Statistic or Category	N = 119%(n)*
Age in years (as of Jan 1, 2019)		
	Mean (SD)	48.5 (14)
	Median	51
	Minimum, Maximum	18, 77
Gender		
	Female	87.4 (104)
	Male	10.9 (13)
	Other/Prefer Not to Answer	1.7 (2)
Race		
	American Indian or Alaskan Native	0.0 ()
	Asian	0.0 ()
	African American	1.7 (2)
	Native Hawaiian or Other Pacific Islander	0.0 ()
	White	96.6 (115)
	Other	1.7 (2)
Ethnicity		
	Hispanic	1.7 (2)
	Not Hispanic	98.3 (117)
Highest level of education completed		
	Less than High School	2.5 (3)
	High School Diploma/Equivalent	5.9 (7)
	Some College	16.0 (19)
	Associates Degree/Technical School	20.2 (24)
	Bachelor's Degree	32.8 (39)
	Graduate of Professional Degree	22.7 (27)
Marital status		
	Single / never married	25.2 (30)
	Married / living as married	58.0 (69)
	Divorced or separated	16.0 (19)
	Widowed / surviving partner	0.8 (1)
	Other	0.0 ()
Do you have children younger than age 18 or other dependents who live with you at home?		
	Yes	17.6 (21)
	No	82.4 (98)
Employment status		
	Employed/Student	46.2 (55)
	Retired	16.0 (19)
	Disabled	29.4 (35)
	Not Currently Employed	8.4 (10)
Time since diagnosis in years (as of Jan 1, 2019)		
	Mean (SD)	11.0 (10.8)
	Median	8
	Minimum, Maximum	<1, 58
Methods ever used to manage PAD symptoms		
	Take prescription pills or tablets	74.8 (89)
	Received extra vaccines	31.9 (38)
	IVIG (Intravenous immunoglobulin infusion) treatment	76.5 (91)
	SCIg (Subcutaneous immunoglobulin infusion) treatments	71.4 (85)
	Bone marrow transplant	0.0 ()
	Changed my lifestyle or exercise routines	57.1 (68)
	Acupuncture, chiropractic adjustments, or dietary supplements	48.7 (58)
	None of the above	0.0 ()
Currently receiving infusions		96.6 (115)
Which option is closest to the way you receive infusions?		(n=115)
	Infusion into the fatty layer under the skin	63.5 (73)
	Infusion into a vein in my arm or hand	24.3 (28)
	Another kind of infusion (for example, through a port or PICC line)	12.2 (14)
Where are your infusions received?		(n=115)
	A nurse comes to my home to administers the infusion	13.9 (16)
	I administer the infusion at home without a nurse	62.6 (72)
	I go to a clinic where a nurse administers my infusion	22.6 (26)
	Other	0.9 (1)
Side effects from last treatment		(n=115)
	Headache	46.1 (53)
	Tiredness / fatigue	73.0 (84)
	Nausea	18.3 (21)
	Rash or skin reaction	23.5 (27)
	Itchiness	22.6 (26)
	Other	15.7 (18)
	No side effects	16.5 (19)

*Unless otherwise noted. †Percentages do not add up to 100% across response categories because respondents were allowed to select multiple answers.

Time since diagnosis of PAD ranged from less than one year to 58 years, with a mean of 11 years since diagnosis and a median time of 8 years. About 77% of participants reported having experience with IVIG for the treatment of PAD. Meanwhile, 71.4% of respondents reported using SCig at some point to treat PAD. Nearly 49% (48.7%) of respondents reported having experience with both IVIG and SCig. Almost all participants (96.6%) reported that they currently receive infusions. Nearly 64% of them using SCig, while about a quarter of the respondents reported using IVIG. Most respondents (62.6%) self-administer their infusion at home.

No respondents were excluded from the final sample based on evidence of nonattention. We also found that no respondent made all treatment choices following the best level of a single attribute. However, 39 patients (32.8%) chose treatment based on the *number of needles* in at least 10 choice questions. Also, 4 patients (3.4%) chose treatment based on *frequency of treatment* in at least 10 choice questions. One patient (0.8%) chose treatment based on *administration time* in at least 10 choice questions, while 9 patients (7.6%) chose treatment based on *duration of side effects* in at least 10 choice questions. Finally, we found that 42.9% and 21.8% of patients incorrectly answered the first and second attribute-comprehension questions, respectively. About 13% of respondents (12.6%) answered both comprehension questions incorrectly. These were the questions meant to test the respondents’ understanding of the DCE task layout. When respondents answered these questions incorrectly, we showed additional information to help them understand the concepts in the comprehension questions. We did not find that respondents who answered these questions incorrectly had different preferences from the rest of the sample (P>0.5).

**Figure 2:**
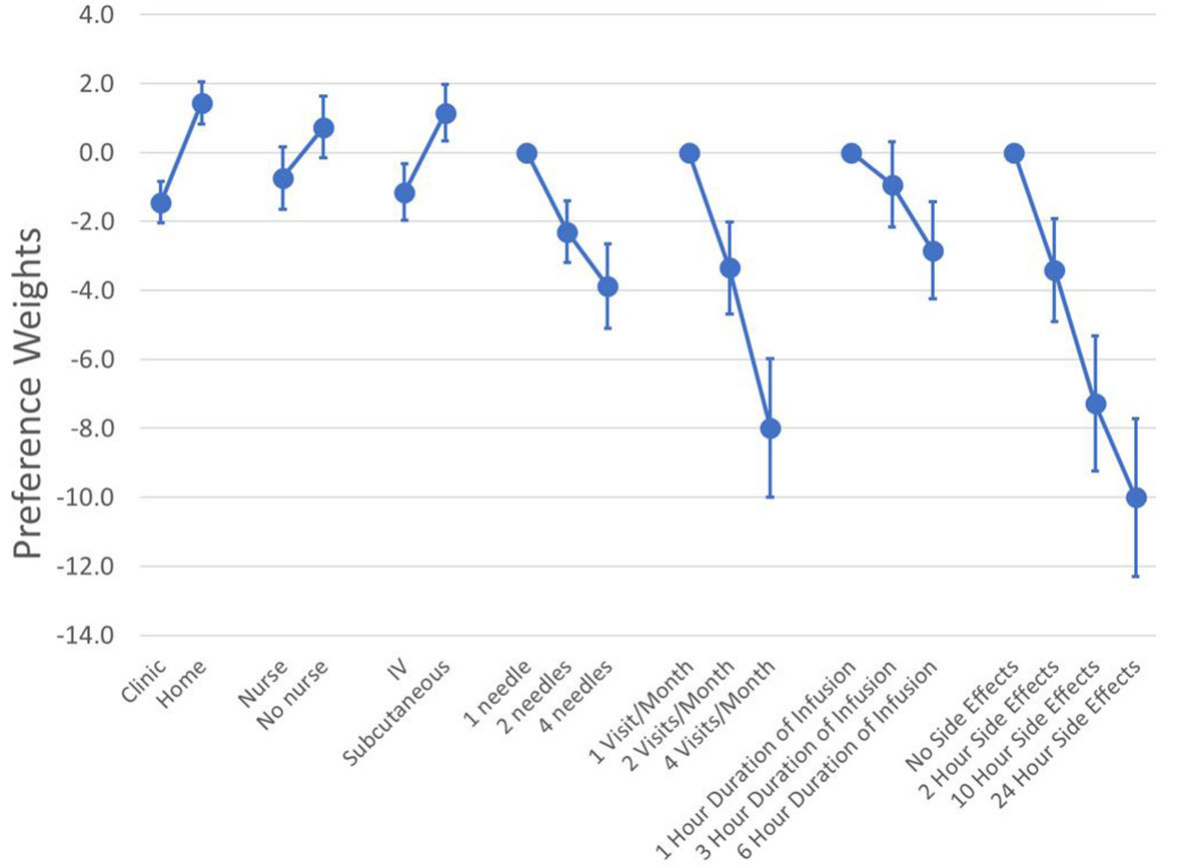
Mean preference weights (N = 119). Log-odds preference weights for all respondents. The absolute value of the weights has no direct meaning. What matters is the relative size of the vertical differences between preferences weights. This is because that vertical distance is correlated with changes in the probability of choice given the attribute change. For example, increasing the duration of infusions from 1 hour to 6 hours reduced the preference weights from 0 to -2.8. Similarly, an increase in the time with side effects from no side effects to 10 hours of side effects decreased the preference weights from 0 to -7.3. This means that the 10-hour increase in the duration of side effects was about 2.6 times (2.6 = -7.3/-2.8) as important as 5-hour increase in the administration time.

We formally evaluated the functional form of the preference model with and without interaction terms between attributes but found that a main-effects specification had the best model fit. **Figure 2** plots the mean preference weights from the RPL model with the full sample and the 95% confidence interval for each attribute level. A table with the raw estimates from the RPL model, including the estimates for the random parameters can be found in **Appendix B**.

While the absolute value of the preference weights is not directly interpretable, higher preference weights indicate greater preference for a treatment with a specific attribute level. To facilitate the interpretation of the preference weights, we normalized all attributes so the most and least preferred attribute levels for duration of side effects had a value of 0 and -10, respectively (see **Figure 2**). All numeric attributes had the expected order of preferences (i.e., better clinical outcomes or less burdensome features were associated with higher preference weights). The differences in the level for route of administration (-1.15 to +1.15), setting (-1.44 to +1.44), and support from a nurse (-0.74 to +0.74) showed some of the smallest overall differences in preference weights. On average, self-administration of SC therapies at home was most preferred by respondents.

**Figure 3:**
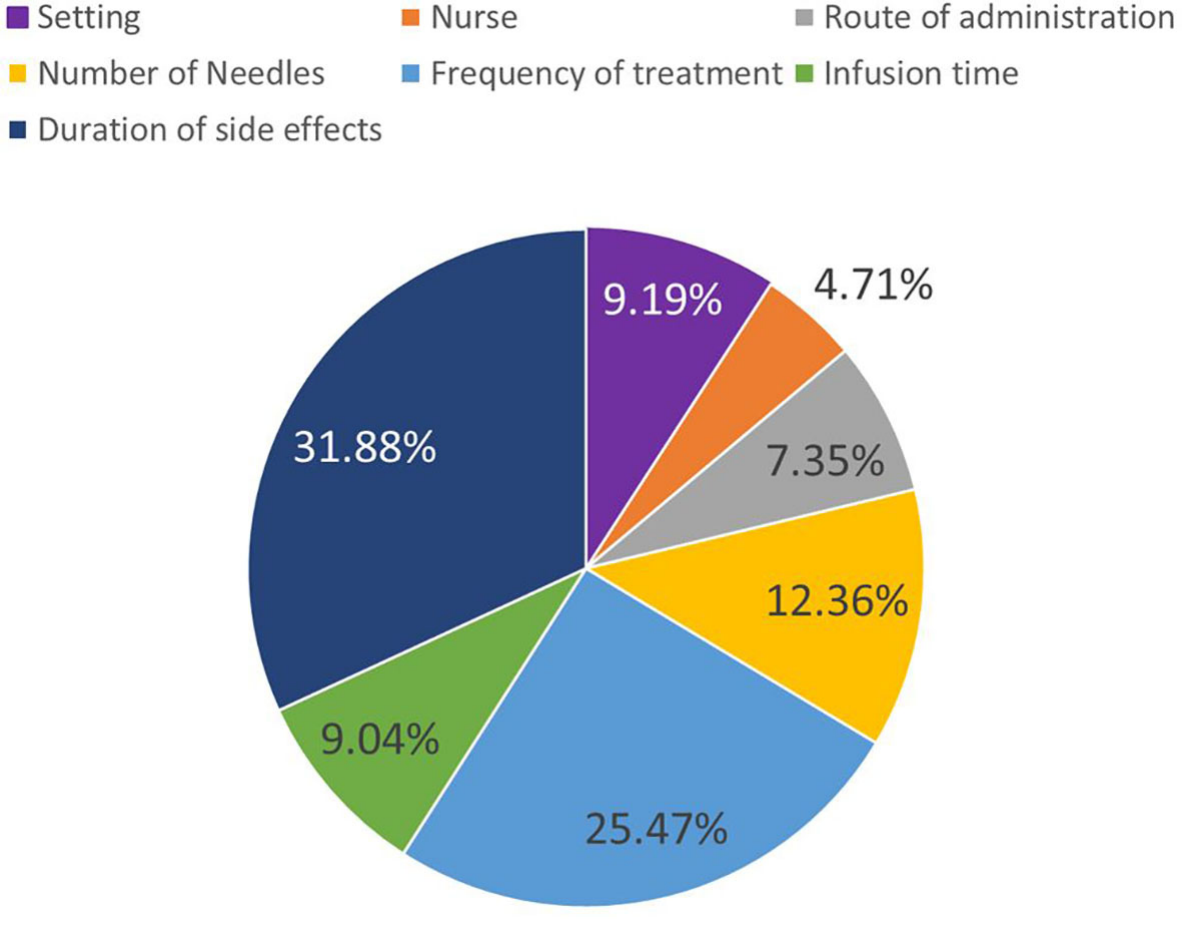
Overall attribute importance. Overall attribute importance weights depict the most influence an attribute change had on treatment choices. This is based on the biggest preference-weight difference within each attribute.

Differences in preference weights between attribute levels are considered the importance of that attribute change. When we consider the most and least preferred levels for an attribute, this difference represents the attributes maximum importance relative to the other features in the study. This is also commonly called overall attribute importance (21). We can normalize that overall attribute importance to evaluate how much each attribute mattered in the DCE tasks presented to respondents. **Figure 3** presents these overall importance values using profile-based normalization (22).

The most important attribute given the range of levels covered in the study was duration of side effects (31.88%), followed by frequency of treatment administration (25.47%), and number of needles required for administration (12.36%). The least important attributes were whether the treatment was administered by a nurse (4.71%), route of administration (7.35%) and treatment setting (9.19%).

Normalized attribute importance does not just indicate the ranking of attributes, but can be used to determine the relative intensity of attribute importance. For example, duration of side effects (31.88%) was approximately 3 times more important than infusion time (9.04%) and treatment setting (9.19%). Meanwhile, frequency of administration (25.47%) was about as important as the three attributes associated with self-administration combined (setting (9.19%), nurse support (4.71%), and route of administration (7.35%) (**Figure 3**).

We evaluated preference heterogeneity based on four patient characteristics: age, years since diagnosis, gender, and previous experience with IVIG and/or SCIg. We failed to reject a hypothesis of equal preferences based on age (respondents above and below the age of 65)(*P-value*=0.83), and gender (female versus males)(*P-value*=0.91). This means that there was not enough information in our data to say that older and younger respondents had different preferences. The same was true of differences between men and women who completed the DCE.

**Figure 4:**
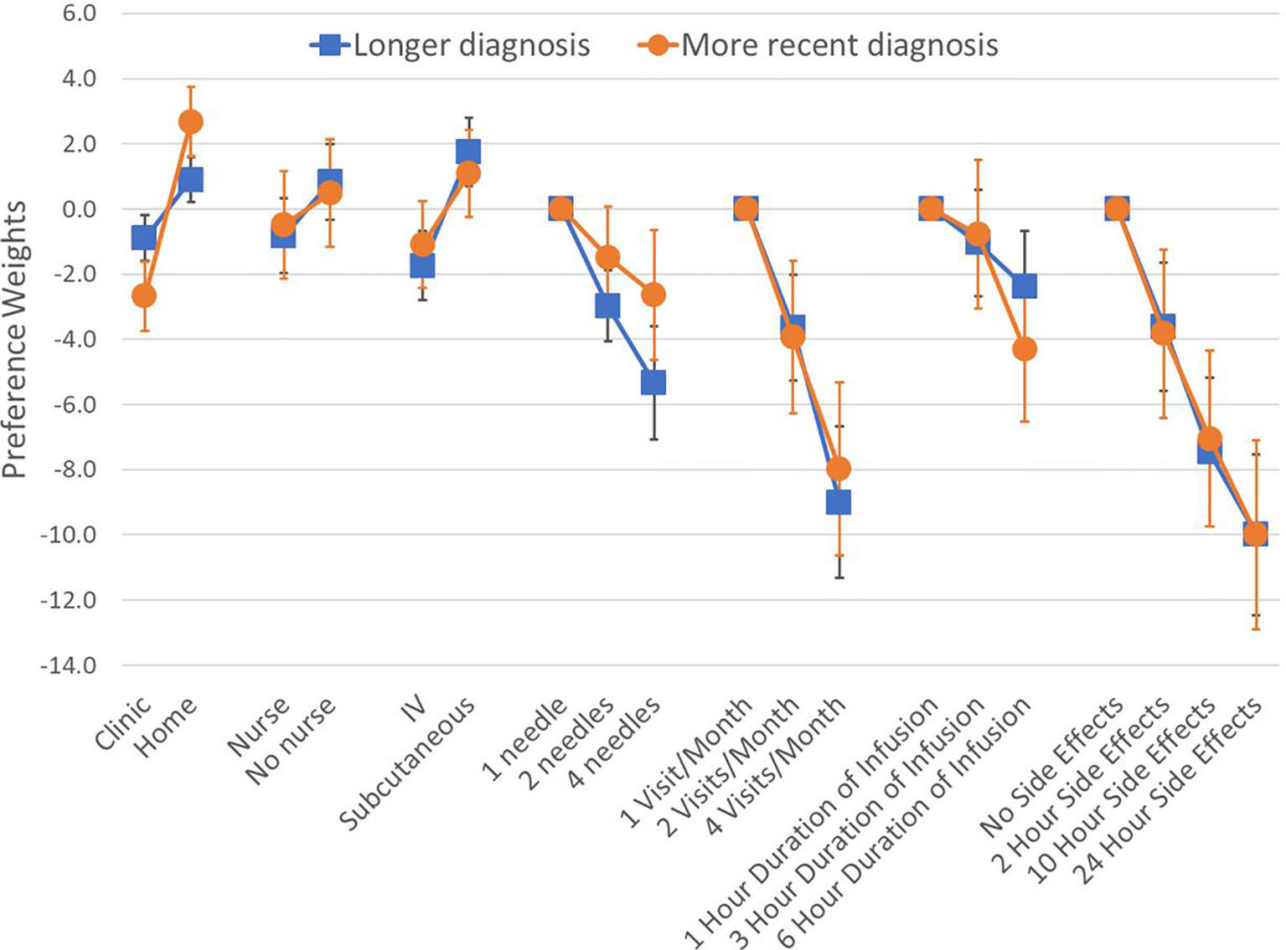
Preference weights by time since diagnosis. Log-odds preference weights for respondents with longer (>8yrs ago) versus more recent diagnosis (<8yrs ago). Lines around each estimate indicate the 95% confidence interval. Results were normalized by overlapping the preference weights for duration of side effects to allow direct comparison between plots. Statistically-significant differences between the groups were found for the administration setting.

**Figure 5:**
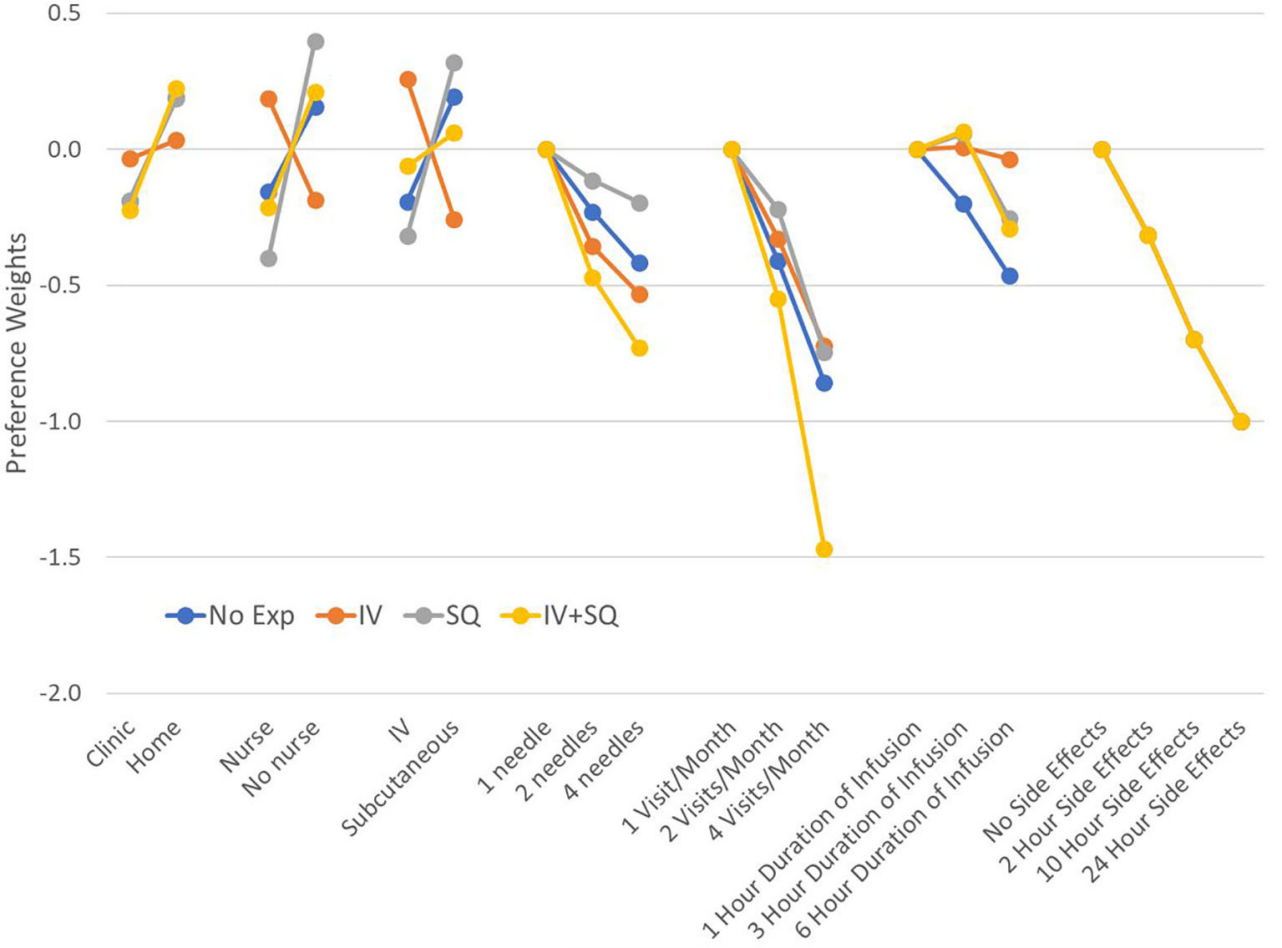
Preference weights by treatment experience. Log-odds preference weights for respondents who reported only using IV therapies (IV), those who reported only experience with (subcutaneous injections), and those who reported experience with both administration options. Results were normalized by overlapping the preference weights for duration of side effects to allow direct comparison between plots. Confidence intervals are not shown to facilitate reading the figure. Estimates and 95% confidence intervals for each subgroup are included in **Appendix B** No exp, No experience with any therapy; IV, Only experience with IVIG; SQ, Only experience with subcutaneous injections; IV+SQ, Experience with both IVIG and subcutaneous injections.

We found that patients with different number of years since diagnosis had different preferences on average. Changes in attribute levels had different impacts on treatment choice across patients who were diagnosed at least 8 years ago (median time since diagnosis in our sample), and those diagnosed more recently (**Figure 4**). Differences in preferences among these subgroups are represented by variations in the vertical distance between point estimates within attributes. Similarly, we found that preferences varied across patients with different treatment experiences (*P-value*<0.001 for IVIG experience, and *P-value*=0.042 for experience with SCIg) (**Figure 5**). As before, all preference weights in each subgroup were normalized so the most and least preferred attribute levels for duration of side effects had a value of 0 and -10, respectively.

We found that across subgroups, respondents generally still preferred to be treated at home. However, respondents who were diagnosed less than 8 years ago were almost three times more concerned about treatment setting than those with longer diagnosis. Regarding treatment experience, results show that respondents who only have IVIG experience prefer using IV therapies and having a nurse administer the treatment. Those who only had experience with SCIg were less concerned about needles and preferred self-administration. Finally, respondents who reported having experience with both IVIG and SCIg appear to be indifferent between the two routes of administration. These respondents also were concerned about the number of needle sticks, side effect duration and number of visits, but preferred self-administration at home. Finally, the patient group with no prior therapy had preferences for SCIg, no nurse and at-home for treatment.

## Discussion

Our study looked to quantify the preferences of patients with PAD based on the factors that most influence their views about treatments. We set out to accomplish this by developing and implementing a DCE. Our results suggest that these patients have well-defined preferences for the attributes we considered in the study.

On average, the patients in our study were primarily concerned about the duration of treatment side effects. Among the process factors considered (excluding health outcomes like side effects), frequency of administration was the most important attribute. We also found that the average respondent seemed to prefer self-administration at home without a nurse. These results are consistent with previously published work on preferences and HRQoL for immunoglobulin therapies (1, 5, 8, 12, 13, 23). However, contrary to Mohamed et al. (12), we did not find significant interaction effects between frequency of administration and duration of administration, duration of side effects, and number or needles. This means respondents did not seem to expect varying levels of disutility from any of these attributes as frequency of treatments increased.

Although generally respondents showed preference for SCIG, the specific dosing given to patients seems to be relevant in an ultimate decision between treatment types. We found that nearly a third of patients chose treatments based on the number of needle sticks in at least 10 of 14 questions. This suggests strong aversion to needles by some respondents. Also, given the levels in our experiment, treatment frequency was about as important as setting, support from a nurse and route of administration. This implies that, on average, patients would be more concerned about the frequency of treatment than the process features associated with IVIG and SCIG. In other words, a less frequent IVIG could look more attractive than a more frequent SCIG.

While respondents in our sample appeared to have well-defined preferences, those preferences were not homogeneous across patients. Both time since diagnosis and treatment experience were correlated with variations in preference weights. Increased time since diagnosis was associated with greater concern with the number of needles required, while experience with a specific treatment type was associated with greater preference for that treatment (IVIG vs. SCIG). The latter may indicate one of two things: 1) patients are already receiving the treatments they want, or 2) they develop affinity for the attributes of the treatments they receive. Either way, our results suggest that at least some patients with PAD may be averse to treatment switching.

The aversion to treatment switching could imply that a formal treatment shared-decision process could facilitate treatment-initiation or treatment-switching discussions and help physicians convey the benefits of different treatment types. Similar efforts have previously shown to have an impact in treatment acceptance and quality of life among patients with common variable immune deficiency. (24) With this in mind, preference-based tools in support of shared decision making could also help improve treatment adherence and outcomes.

It is worth noting some key limitations of our study. The survey elicits preferences between hypothetical treatment options. The recorded choices do not carry the same consequences as real-world treatment decisions. While the choices elicited here might be different from those made in a clinical context, the study team followed best practices in survey research to make the questions consequential and to induce preference-revealing answers (25). Another important limitation is that the relative importance of the attributes elicited through the DCE are conditional on the attributes and attribute levels included in the study. That said, these attributes and levels were defined with direct patient input and in consultation with clinical experts. Finally, while the characteristics of survey respondents were largely consistent with samples from previous studies conducted in this population (9, 24), our sampling framework does not guarantee that our preference estimates are representative of the broader PAD patient population. Despite potential issues with the representativeness of the study sample, the identified variations in preferences suggest there are systematic differences in the acceptability of the tradeoffs implied by specific treatment options.

## Conclusions

The majority of patients with PAD in our study wanted to be treated at home, but we found that setting or route of administration represent a relatively small part of patients’ preferences for treatments, so treatment dosing could overcome the benefits of treatment route of administration. We also found that patient preferences for treatments were not homogeneous across patients. Treatment experience can be associated with preferences for IV administration with a nurse. These heterogeneous views on the relative importance of aspects of treatments, suggests that a formal shared decision making process could have an important role in improving patient care, particularly if patients indeed are adapting to therapies that may result in unnecessary treatment burden. Such a proposal is not new (9, 26) and instruments like the one developed for this study could be adjusted to help document patients’ views in a clinical setting. The information collected through such a preference-elicitation tool could support open discussions around the tradeoffs that patients are willing to accept between treatment aspects, and potentially help minimize HRQoL impacts of treatments by adequately matching patients’ preferences and treatment options.

## Data Availability Statement

The raw data supporting the conclusions of this article will be made available by the authors, without undue reservation.

## Ethics Statement 

The studies involving human participants were reviewed and approved by Duke University Institutional Review Board. Written informed consent for participation was not required for this study in accordance with the national legislation and the institutional requirements.

## Author Contributions

JG led the study design, survey development, data analysis and the drafting the manuscript. MB contributed to the study design, survey development, data analysis and drafting of the manuscript. AF contributed to the study design, survey development, data analysis, and provided critical input to the development of the manuscript. CR contributed to the study design, survey development, data analysis and provided critical input to the development of the manuscript. All authors contributed to the article and approved the submitted version.

## Funding

Financial support for this study was provided in part by Grifols SSNA. The funding agreement ensured the authors’ independence in designing the study, interpreting the data, writing, and publishing the report.

## Conflict of Interest

MB has been a consultant and speaker for Grifols SSNA and Green Cross DSMB. MB is also a consultant and advisor to the Immune Deficiency Foundation. Also, MCR is currently an employee of Grifols.

The remaining authors declare that the research was conducted in the absence of any commercial or financial relationships that could be construed as a potential conflict of interest.

## Publisher’s Note

All claims expressed in this article are solely those of the authors and do not necessarily represent those of their affiliated organizations, or those of the publisher, the editors and the reviewers. Any product that may be evaluated in this article, or claim that may be made by its manufacturer, is not guaranteed or endorsed by the publisher.
