# Supplementary material for: Primary Immune Deficiency: Patients’ Preferences for Replacement Immunoglobulin Therapy
Source: Front Immunol. 2022 Feb 4;13:827305. doi: 10.3389/fimmu.2022.827305 (PMC8854788; doi:10.3389/fimmu.2022.827305)
Supplement: Supplementary file 1 [file DataSheet_1.docx]

**Appendix A.**

**Experimental design**

We generated two D-efficient fractional-factorial designs using an algorithm developed using SAS® version 9.4 (Cary, NC). The first design included 60 questions blocked in 15 sets of 4 questions each. The second design also included 60 questions, but was blocked to create 6 sets of 10 questions each. Each respondent was shown one set from each design for a total of 14 choice questions. The order of the questions within and across sets were randomized to address order effects. Three attributes (route of administration, treatment setting, and support from a nurse) were combined under the label “How the attribute is administered” to facilitate the exclusion of nonsensical attribute combinations (e.g., self-administration at a clinic with a nurse).

Based on feedback obtained from respondents during the pretest interviews, we were concerned that the effect size of preferences for treatment frequency, infusion time, and duration of side effects was significantly larger than the effect of route of administration, clinical setting, and support from a nurse. Thus, the first D-efficient designs forced the levels of treatment frequency, infusion time, and duration of side effects to be the same across alternatives. This motivated respondents to focus on the differences in the remaining attributes, increasing the information obtained on preferences for attributes with smaller effect sizes.

**Internal validity of DCE data**

These checks included identifying response nonvariation in preferences (straight lining), attribute dominance, and the effect of attribute-comprehension questions asked before the DCE was completed. Mechanisms to address any observed quality issues were outlined prior to analysis.

Verification of response nonvariation allows us to evaluate the extent to which respondents were paying attention to the attribute levels shown in the choice questions. The experimental design created question such that the better alternative was extremely unlikely to be in the same position (i.e., Treatment A or Treatment B) in all questions. Thus, patients who always chose one of these alternatives were considered to be nonattentive and were excluded from the study sample.

Attribute dominance refers to the consistent selection of treatment alternatives based solely on one attribute. This could reflect strong preferences for a specific attribute or a simplifying heuristic to aid in the completion of the survey. Without auxiliary information, it is not possible to determine which of the two is driving such choices. Thus, we retained respondents who dominated on a specific attribute, assuming these choices represented true preferences.

Finally, two attribute-comprehension questions were asked before the respondents answered the DCE questions. If respondents answered any of these questions incorrectly, they were shown a follow-up screen explaining the correct response. We addressed the impact of respondents who missed one or more of the comprehension questions by testing whether their preferences varied systematically from those who answered the questions correctly.

**Analysis**

The primary analysis of the DCE data used an uncorrelated random-parameters logit (RPL) model. The RPL model leverages repeated observations from each respondent to estimate a distribution of individual-level preferences. All attribute levels were included in the model specification as categorical variables, either with dummy-coding or effect-coding, and preferences were assumed to follow a normal distribution across respondents.

Finally, we used a scale-adjusted RPL model to test for differences between specific pairs of subgroups defined by individual characteristics collected in our survey. The scale-adjusted RPL follows Hensher et al. (Hensher et al., 2008) as a way to estimate group-specific variance to properly evaluate differences in preferences across subgroups of interest.

The scale-adjusted RPL estimates group-specific variance. The group-specific variance estimate allows testing for differences in the preference estimates for each cohort after controlling for systematic differences in scale/variance heterogeneity.

An indicator variable for one of the subgroups of interest was interacted with the variables for each attribute level. These interactions were included in the model specification to represent the difference in preferences between a baseline subgroup and the subgroup flagged by the indicator variable. A joint test of significance (Wald chi-square test) of the interaction terms was used to determine whether preferences between the two subgroups were statistically significantly different.

Reference

Hensher, David A., John M. Rose, and William H. Greene. "Combining RP and SP data: biases in using the nested logit ‘trick’–contrasts with flexible mixed logit incorporating panel and scale effects." Journal of Transport Geography 16.2 (2008): 126-133. https://doi.org/10.1016/j.jtrangeo.2007.07.001
