# Supplementary material for: Primary Immune Deficiency: Patients’ Preferences for Replacement Immunoglobulin Therapy
Source: Front Immunol. 2022 Feb 4;13:827305. doi: 10.3389/fimmu.2022.827305 (PMC8854788; doi:10.3389/fimmu.2022.827305)
Supplement: Supplementary file 2 [file DataSheet_2.docx]

**Appendix B. Random-parameters logit results**

1. **Overall preference results**

|  | **Mean** | **SE** |  |
| --- | --- | --- | --- |
| Clinic | -0.439 | 0.094 |  |
| Home | 0.439 | 0.094 |  |
|  |  |  |  |
| Nurse | -0.226 | 0.141 |  |
| No nurse | 0.226 | 0.141 |  |
|  |  |  |  |
| IV | -0.352 | 0.128 |  |
| Subcutaneous | 0.352 | 0.128 |  |
|  |  |  |  |
| 1 needle | 0.000 | 0.000 |  |
| 2 needles | -0.702 | 0.140 |  |
| 4 needles | -1.183 | 0.191 |  |
|  |  |  |  |
| 1 Visit/Month | 0.000 | 0.000 |  |
| 2 Visits/Month | -1.021 | 0.208 |  |
| 4 Visits/Month | -2.437 | 0.314 |  |
|  |  |  |  |
| 1 Hour Duration | 0.000 | 0.000 |  |
| 3 Hour Duration | -0.282 | 0.193 |  |
| 6 Hour Duration | -0.865 | 0.218 |  |
|  |  |  |  |
| No Side Effects | 0.000 | 0.000 |  |
| 2 Hour Side Effects | -1.038 | 0.232 |  |
| 10 Hour Side Effects | -2.222 | 0.306 |  |
| 24 Hour Side Effects | -3.050 | 0.356 |  |
|  | **SD** | **SE** |  |
| Clinic | 0.658 | 0.131 |  |
| Home | 0.658 | 0.131 |  |
|  |  |  |  |
| Nurse | 1.191 | 0.187 |  |
| No nurse | 1.191 | 0.187 |  |
|  |  |  |  |
| IV | 1.340 | 0.207 |  |
| Subcutaneous | 1.340 | 0.207 |  |
|  |  |  |  |
| 1 needle | 0.000 | 0.000 |  |
| 2 needles | -0.326 | 0.264 |  |
| 4 needles | 0.729 | 0.239 |  |
|  |  |  |  |
| 1 Visit/Month | 0.000 | 0.000 |  |
| 2 Visits/Month | -0.100 | 0.344 |  |
| 4 Visits/Month | 1.447 | 0.256 |  |
|  |  |  |  |
| 1 Hour Duration | 0.000 | 0.000 |  |
| 3 Hour Duration | 0.301 | 0.445 |  |
| 6 Hour Duration | 0.587 | 0.249 |  |
|  |  |  |  |
| No Side Effects | 0.000 | 0.000 |  |
| 2 Hour Side Effects | -0.540 | 0.312 |  |
| 10 Hour Side Effects | -0.916 | 0.359 |  |
| 24 Hour Side Effects | 1.587 | 0.302 |  |

IV = Intravenous.

**B. Results by time since diagnosis**

|  | **Older diagnosis** | |  | **More recent diagnosis** | |
| --- | --- | --- | --- | --- | --- |
|  | **Mean** | **SE** |  | **Mean** | **SE** |
| Clinic | -0.274 | 0.108 |  | -0.552 | 0.111 |
| Home | 0.274 | 0.108 |  | 0.552 | 0.111 |
|  |  |  |  |  |  |
| Nurse | -0.253 | 0.179 |  | -0.100 | 0.173 |
| No nurse | 0.253 | 0.179 |  | 0.100 | 0.173 |
|  |  |  |  |  |  |
| IV | -0.531 | 0.164 |  | -0.226 | 0.141 |
| Subcutaneous | 0.531 | 0.164 |  | 0.226 | 0.141 |
|  |  |  |  |  |  |
| 1 needle | 0.000 | 0.000 |  | 0.000 | 0.000 |
| 2 needles | -0.908 | 0.171 |  | -0.307 | 0.162 |
| 4 needles | -1.631 | 0.269 |  | -0.543 | 0.209 |
|  |  |  |  |  |  |
| 1 Visit/Month | 0.000 | 0.000 |  | 0.000 | 0.000 |
| 2 Visits/Month | -1.112 | 0.253 |  | -0.812 | 0.245 |
| 4 Visits/Month | -2.746 | 0.362 |  | -1.644 | 0.279 |
|  |  |  |  |  |  |
| 1 Hour Duration | 0.000 | 0.000 |  | 0.000 | 0.000 |
| 3 Hour Duration | -0.318 | 0.255 |  | -0.160 | 0.239 |
| 6 Hour Duration | -0.729 | 0.268 |  | -0.890 | 0.232 |
|  |  |  |  |  |  |
| No Side Effects | 0.000 | 0.000 |  | 0.000 | 0.000 |
| 2 Hour Side Effects | -1.103 | 0.306 |  | -0.788 | 0.272 |
| 10 Hour Side Effects | -2.278 | 0.354 |  | -1.453 | 0.283 |
| 24 Hour Side Effects | -3.051 | 0.384 |  | -2.060 | 0.306 |
|  | **SD** | **SE** |  |  |  |
| Clinic | 0.531 | 0.101 |  |  |  |
| Home | 0.531 | 0.101 |  |  |  |
|  |  |  |  |  |  |
| Nurse | 1.087 | 0.181 |  |  |  |
| No nurse | 1.087 | 0.181 |  |  |  |
|  |  |  |  |  |  |
| IV | 1.125 | 0.149 |  |  |  |
| Subcutaneous | 1.125 | 0.149 |  |  |  |
|  |  |  |  |  |  |
| 1 needle | 0.000 | 0.000 |  |  |  |
| 2 needles | 0.119 | 0.186 |  |  |  |
| 4 needles | 0.818 | 0.227 |  |  |  |
|  |  |  |  |  |  |
| 1 Visit/Month | 0.000 | 0.000 |  |  |  |
| 2 Visits/Month | 0.352 | 0.246 |  |  |  |
| 4 Visits/Month | 1.335 | 0.245 |  |  |  |
|  |  |  |  |  |  |
| 1 Hour Duration | 0.000 | 0.000 |  |  |  |
| 3 Hour Duration | 0.162 | 0.213 |  |  |  |
| 6 Hour Duration | -0.137 | 0.262 |  |  |  |
|  |  |  |  |  |  |
| No Side Effects | 0.000 | 0.000 |  |  |  |
| 2 Hour Side Effects | -0.442 | 0.248 |  |  |  |
| 10 Hour Side Effects | 0.605 | 0.272 |  |  |  |
| 24 Hour Side Effects | 1.483 | 0.323 |  |  |  |
| Scale control for more recent diagnosis | 0.137 | 0.066 |  |  |  |

IV = Intravenous.

**C. Results by treatment experience**

|  | **No Experience** | |  | **IV** | |  | **SQ** | |  | **IV+SQ** | |
| --- | --- | --- | --- | --- | --- | --- | --- | --- | --- | --- | --- |
|  | **Mean** | **SE** |  | **Mean** | **SE** |  | **Mean** | **SE** |  | **Mean** | **SE** |
| Clinic | -0.563 | 0.100 |  | -0.102 | 0.263 |  | -0.558 | 0.272 |  | -0.660 | 0.194 |
| Home | 0.563 | -0.100 |  | 0.102 | -0.263 |  | 0.558 | -0.272 |  | 0.660 | 0.194 |
|  |  |  |  |  |  |  |  |  |  |  |  |
| Nurse | -0.463 | 0.160 |  | 0.552 | 0.437 |  | -1.179 | 0.429 |  | -0.627 | 0.318 |
| No nurse | 0.463 | -0.160 |  | -0.552 | -0.437 |  | 1.179 | -0.429 |  | 0.627 | 0.318 |
|  |  |  |  |  |  |  |  |  |  |  |  |
| IV | -0.568 | 0.136 |  | 0.759 | 0.369 |  | -0.941 | 0.401 |  | -0.182 | 0.269 |
| Subcutaneous | 0.568 | -0.136 |  | -0.759 | -0.369 |  | 0.941 | -0.401 |  | 0.182 | 0.269 |
|  |  |  |  |  |  |  |  |  |  |  |  |
| 1 needle | 0.000 | 0.000 |  | 0.000 | 0.000 |  | 0.000 | 0.000 |  | 0.000 | 0.000 |
| 2 needles | -0.678 | 0.174 |  | -1.050 | 0.450 |  | -0.337 | 0.475 |  | -1.386 | 0.342 |
| 4 needles | -1.232 | 0.230 |  | -1.569 | 0.608 |  | -0.578 | 0.623 |  | -2.146 | 0.492 |
|  |  |  |  |  |  |  |  |  |  |  |  |
| 1 Visit/Month | 0.000 | 0.000 |  | 0.000 | 0.000 |  | 0.000 | 0.000 |  | 0.000 | 0.000 |
| 2 Visits/Month | -1.214 | 0.265 |  | -0.966 | 0.684 |  | -0.652 | 0.709 |  | -1.618 | 0.498 |
| 4 Visits/Month | -2.529 | 0.350 |  | -2.130 | 0.842 |  | -2.200 | 0.855 |  | -4.330 | 0.612 |
|  |  |  |  |  |  |  |  |  |  |  |  |
| 1 Hour Duration | 0.000 | 0.000 |  | 0.000 | 0.000 |  | 0.000 | 0.000 |  | 0.000 | 0.000 |
| 3 Hour Duration | -0.592 | 0.263 |  | 0.022 | 0.677 |  | 0.170 | 0.708 |  | 0.192 | 0.469 |
| 6 Hour Duration | -1.367 | 0.292 |  | -0.106 | 0.723 |  | -0.748 | 0.747 |  | -0.855 | 0.484 |
|  |  |  |  |  |  |  |  |  |  |  |  |
| No Side Effects | 0.000 | 0.000 |  | 0.000 | 0.000 |  | 0.000 | 0.000 |  | 0.000 | 0.000 |
| 2 Hour Side Effects | -0.925 | 0.211 |  | -0.925 | 0.211 |  | -0.925 | 0.211 |  | -0.925 | 0.211 |
| 10 Hour Side Effects | -2.059 | 0.244 |  | -2.059 | 0.244 |  | -2.059 | 0.244 |  | -2.059 | 0.244 |
| 24 Hour Side Effects | -2.952 | 0.298 |  | -2.952 | 0.298 |  | -2.952 | 0.298 |  | -2.952 | 0.298 |
|  | **SD** | **SE** |  |  |  |  |  |  |  |  |  |
| Clinic | 0.583 | 0.172 |  |  |  |  |  |  |  |  |  |
| Home | 0.583 | 0.172 |  |  |  |  |  |  |  |  |  |
|  |  |  |  |  |  |  |  |  |  |  |  |
| Nurse | 1.904 | 0.303 |  |  |  |  |  |  |  |  |  |
| No nurse | 1.904 | 0.303 |  |  |  |  |  |  |  |  |  |
|  |  |  |  |  |  |  |  |  |  |  |  |
| IV | 1.861 | 0.280 |  |  |  |  |  |  |  |  |  |
| Subcutaneous | 1.861 | 0.280 |  |  |  |  |  |  |  |  |  |
|  |  |  |  |  |  |  |  |  |  |  |  |
| 1 needle | 0.000 | 0.000 |  |  |  |  |  |  |  |  |  |
| 2 needles | 0.219 | 0.366 |  |  |  |  |  |  |  |  |  |
| 4 needles | 1.535 | 0.436 |  |  |  |  |  |  |  |  |  |
|  |  |  |  |  |  |  |  |  |  |  |  |
| 1 Visit/Month | 0.000 | 0.000 |  |  |  |  |  |  |  |  |  |
| 2 Visits/Month | 0.855 | 0.399 |  |  |  |  |  |  |  |  |  |
| 4 Visits/Month | 2.642 | 0.507 |  |  |  |  |  |  |  |  |  |
|  |  |  |  |  |  |  |  |  |  |  |  |
| 1 Hour Duration | 0.000 | 0.000 |  |  |  |  |  |  |  |  |  |
| 3 Hour Duration | 0.715 | 0.539 |  |  |  |  |  |  |  |  |  |
| 6 Hour Duration | 0.977 | 0.463 |  |  |  |  |  |  |  |  |  |
|  |  |  |  |  |  |  |  |  |  |  |  |
| No Side Effects | 0.000 | 0.000 |  |  |  |  |  |  |  |  |  |
| 2 Hour Side Effects | 0.671 | 0.298 |  |  |  |  |  |  |  |  |  |
| 10 Hour Side Effects | 0.754 | 0.209 |  |  |  |  |  |  |  |  |  |
| 24 Hour Side Effects | 1.613 | 0.349 |  |  |  |  |  |  |  |  |  |
| Scale control for IV-experience group | 0.009 | 0.264 |  |  |  |  |  |  |  |  |  |
| Scale control for SQ-experience group | 0.421 | 0.295 |  |  |  |  |  |  |  |  |  |

No experience = No experience with any therapy; IV = Only experience with IVIG; SQ = Only experience with subcutaneous injections, IV+SQ = Experience with both IVIG and subcutaneous injections.

SD = Standard deviation; SE = Standard Error;
